# *COL6A3* polymorphisms were associated with lung cancer risk in a Chinese population

**DOI:** 10.1186/s12931-019-1114-y

**Published:** 2019-07-08

**Authors:** Ying Duan, Gaowen Liu, Yao Sun, Jiamin Wu, Zichao Xiong, Tianbo Jin, Mingwei Chen

**Affiliations:** 1 Department of Respiratory Medicine, The First Affiliated Hospital of School of Medicine of Xi’an Jiao Tong University, #277 Yanta West Road, Xi’an, 710061 Shaanxi China; 2 Xianyang Central Hospital, Xianyang, 712000 Shaanxi China; 3 Key Laboratory of Resource Biology and Biotechnology in Western China (Northwest University), Ministry of Education, Xi’an, 710069 Shaanxi China

**Keywords:** Lung cancer, Susceptibility, Agena MassARRAY technology, Case-control study, *COL6A3*, Single nucleotide polymorphisms

## Abstract

**Background:**

Lung cancer is one of the leading cause of cancer-related death in the world. Recently, many clinical researches have reported that *COL6A3* had strong role in many diseases. The aim of this study was to evaluate the association between single nucleotide polymorphisms (SNPs) in *COL6A3* and lung cancer susceptibility.

**Method:**

Eight variants in *COL6A3* were genotyped in a Chinese Han population including 510 cases and 495 controls using Agena MassARRAY. Genetic models and haplotype analyses were used to calculate the association between *COL6A3* SNPs and lung cancer risk. And we assessed the relative risk by the odds ratio (OR) and 95% confidence interval (CI).

**Results:**

In our results, we observed that rs115510139 was linked to an increased risk of lung cancer in the codominant (adjusted OR = 1.61, 95%CI: 1.14–2.27, *p* = 0.007), dominant (adjusted OR = 1.36, 95%CI: 1.02–1.83, *p* = 0.037), recessive (adjusted OR = 1.41, 95%CI: 1.07–1.85, *p* = 0.015), and log-additive (adjusted OR = 1.27, 95%CI: 1.07–1.51, *p* = 0.006) models. After gender stratification analysis, we found that rs115510139, rs3736341 and rs12052971 were significant in males but were non-significant in females. Rs115510139 also can increase the risk of lung cancer in the population of age less than 61 years. When analyzed for the association with lung squamous carcinoma, rs13032404, rs115510139 and rs3736341 were related to the risk of lung cancer.

**Conclusions:**

Our findings indicated potential associations between *COL6A3* polymorphisms and lung cancer risk, which may contribute to the identification of lung cancer patients in a Chinese population.

**Electronic supplementary material:**

The online version of this article (10.1186/s12931-019-1114-y) contains supplementary material, which is available to authorized users.

## Background

Globally, lung cancer is one of the most common types of incident cancer and the leading cause of cancer-related death [1, 2]. It is mainly divided into small cell lung cancer (SCLC) and non-small cell lung cancer (NSCLC), whose mainly clinical manifestation is NSCLC. According to the morphological characteristics of tumor cells under the microscope, NSCLC usually treated with platinum-based chemotherapy [3] was consisted of squamous cell carcinoma, adenocarcinoma, large-cell carcinoma and so on. In addition, lung cancer is the main reason of cancer death for men and the second leading cause of cancer death for women (second only to breast cancer). Every year, there were about 1.8 million new cases diagnosed with lung cancer and about 1.6 million lung cancer deaths estimated in 2012 [4]. Besides, it is well known that cancer is difficult to cure and has a poor prognosis with a 5-year survival rate of lung cancer populations depending on stage and regional differences [2, 5]. In China, lung cancer is also the most common incident cancer and the main cause of cancer death. It was estimated that about 733.3/100,000 cases were diagnosed with lung cancer and 610.5/100,000 died of lung cancer in 2015 [2]. Moreover, new cases and mortality rates of lung cancer have been increasing year by year, so, it is urgent to study the pathogenesis of lung cancer to prevent and treat it better in the future.

But, chemotherapy resistance makes the treatment of lung cancer patients less effective. According to relevant research, the occurrence of lung cancer may have genetic predisposition [6]. Recently, large-scale genome wide association studies were adopted to identify lung cancer susceptibility genes located in chromosomes 5p15.33, 6p21, 6q23–25, 15q24–25.1, and 13q31.3 [7], suggesting that the regions of these chromosomes were associated with the risk of lung cancer, especially in never-smokers.

The *COL6A3* (Collagen Type VI Alpha 3 Chain) gene contains 44 exons and is located in chromosome 2q37.3. *COL6A3*, encoding the a3(VI) chain, contains two C terminal Von Willebrand factor type A-like domains, subdomains similar to type III fibronectin repeats, and Kunitz protease inhibitors as well as 6–10 N-terminal Von Willebrand factor type A-like domains, thus contributing to most of the amino-terminal globular domain of the collagen VI heterotrimer. This protein is an extracellular matrix protein found in most tissues and its absence or aberrant formation can result in many diseases, such as Congenital Muscular Dystrophies (CMDs) [8].

In our study, we found that the region in chromosome 2q37.3 was also related to the risk of lung cancer. In addition, our research firstly discussed that *COL6A3* gene was associated with lung cancer risk. We aimed to analyze the genetic association of *COL6A3* and lung cancer risk among a Chinese population of Shaanxi Han.

## Methods and materials

### Study subjects

We performed the study in accordance with the Declaration of Helsinki. And the protocol was approved by the Ethics Committee of Shaanxi Provincial Cancer Hospital. Using a case-control design, 510 inpatients with lung cancer and 495 controls were enrolled. All patients were recruited between March 2017 and October 2018 from the Shaanxi Provincial Cancer Hospital (Xi’an City, Shaanxi, China) and they were diagnosed with lung cancer, and they had no history of any other cancers. Healthy individuals without any diseases collected from health examination center were used as controls. All patients and controls were a Chinese Han population. And written informed consent was obtained from all individual participants. In total, we collected 1005 peripheral venous blood samples using vacutainer tubes containing EDTA, which were then stored at − 80 °C refrigerator for DNA extraction experiments.

### SNP selection and genotyping

In total, we successfully chose eight variants (rs1050785, rs7436, rs13032404, rs115510139, rs2645765, rs3736341, rs12052971, rs6720283) in the global population of the 1000 Genome Projects (http://www.internationalgenome.org/). Each SNP had a minor allele frequency (MAF) > 5%. RegulomeDB (http://www.regulomedb.org/) and HaploReg (https://pubs.broadinstitute.org/mammals/haploreg/haploreg.php) were utilized to predict SNP function. According to the manufacturer’s instructions of the GoldMag-Mini Whole Blood Genomic DNA Purification Kit (GoldMag. Co. Ltd., Xi’an, China), we extracted genomic DNA from the blood samples. Then, DNA concentration and purity were checked using a spectrophotometer (NanoDrop 2000; Thermo Fisher Scientific, Waltham, MA, USA). Primers for amplification and extension of SNPs were designed by using the Agena Bioscience Assay Design Suite V2.0 software (https://agenacx.com/online-tools/) (Additional file 4: Table S1). Then, we performed SNP genotyping with the Agena MassARRAY platform with iPLEX gold chemistry (Agena Bioscience, San Diego, CA, USA), and managed and analyzed data with Agena Bioscience TYPER, Version 4.0 [9].

### Analysis of *COL6A3* expression

We observed the differences in *COL6A3* expression between normal lung tissues and lung cancer tissues based on the UALCAN database (http://ualcan.path.uab.edu/index.html), which is an interactive web resource for analyzing the transcriptome data of cancer. And the Kaplan-Meier Plotter database (http://kmplot.com/analysis/index.php?p=background) included the information downloaded from GEO (Gene Expression Omnibus), EGA (European Genome-phenome Archive) and TCGA (The Cancer Genome Atlas) database was used to analyze the prognostic value of a specific gene. The correlation between the expression of *COL6A3* in lung cancer and the overall survival rate was displayed by a Kaplan-Meier survival plot, the hazard ratio (HR) with 95%CIs and log-rank *p* value.

### Statistical analysis

The Student’s t-test and Pearson’s test were applied to evaluate differences in the distribution of age and gender between two groups (cases and controls), respectively. The genotype frequencies among the controls were evaluated departure from Hardy-Weinberg Equilibrium (HWE). And we calculated the association between SNPs and lung cancer risk based on the four model analyses (codominant, dominant, recessive, and log-additive) using logistic regression analysis provided by the PLINK software (version 1.07) [10]. Additionally, Haploview software (version 4.2) was used to generate a linkage disequilibrium (LD) map to observe the degree of linkage between these eight SNPs. All *p*-values were two-tailed and *p*-values less than 0.05 were considered statistically significant.

## Results

### Characteristics of cases and controls

The case group consisted of 510 inpatients with lung cancer. Three hundred forty-six were males and 149 were females, with mean age 60.78 ± 9.958 years. And 495 volunteers (355 males and 155 females) were used as the controls, with mean age 61.94 ± 7.723 years. The basic information of eight *COL6A3* polymorphisms was displayed in Table 1. The genotype distribution of all eight SNPs in the control group was in accordance with HWE (*p* > 0.05). The frequency distribution of allele “C” of rs115510139 was significantly different between cases and controls (*p* = 0.006), from which we found it to be associated with an increased risk of lung cancer (OR = 1.28, 95%CI: 1.08–1.53) in the Chinese Han population. The RegulomeDB Score and HaploReg were used to evaluate the function of the SNPs listed in Additional file 8: Table S5.

**Table 1:** Basic information and allele frequencies of the SNPs in *COL6A3*

SNP	Chr	Gene	Alleles A < B	Role	MAF (A)		HWE *p-*value	OR (95%CI)	*P* ^*^
					Case	Control			
rs1050785	2q37.3	*COL6A3*	G/T	3′-UTR	0.469	0.492	0.654	0.91 (076–1.09)	0.296
rs7436	2q37.3	*COL6A3*	A/T	3′-UTR	0.171	0.182	0.764	0.92 (0.73–1.16)	0.495
rs13032404	2q37.3	*COL6A3*	A/G	Intron	0.387	0.367	0.148	1.09 (0.91–1.30)	0.359
rs115510139	2q37.3	*COL6A3*	A/T	Intron	0.558	0.496	0.241	1.28 (1.08–1.53)	0.006
rs2645765	2q37.3	*COL6A3*	A/G	Intron	0.280	0.297	0.386	0.92 (0.76–1.12)	0.393
rs3736341	2q37.3	*COL6A3*	C/T	Intron	0.247	0.273	0.131	0.87 (0.71–1.07)	0.195
rs12052971	2q37.3	*COL6A3*	A/G	Intron	0.250	0.249	0.103	1.00 (0.82–1.23)	0.976
rs6720283	2q37.3	*COL6A3*	A/G	Intron	0.459	0.447	0.466	1.05 (0.88–1.25)	0.606

*95%CI* 95% confidence interval, *HWE* Hardy-Weinberg equilibrium, *MAF* Minor allele frequency, *OR* Odds ratio, *SNP* Single-nucleotide polymorphism

p^*^: Calculated by Pearson χ^2^ test

### Genetic model analysis between *COL6A3* variants and lung cancer risk

To further explore the correlations between *COL6A3* variants and lung cancer risk, genetic models (codominant, dominant, recessive, and additive) were applied to this study (Table 2). We observed that the genotypes “T/A-T/T” of rs115510139 were linked to an increased risk of lung cancer than genotype “A/A” in the dominant model with or without adjustment for gender and age (OR = 1.36, 95%CI: 1.01–1.82, *p* = 0.040; adjusted OR = 1.36, 95%CI: 1.02–1.83, *p* = 0.037). Genotype “TT” of rs115510139 had an increased risk of lung cancer in the codominant (OR = 1.60, 95%CI: 1.13–2.25, *p* = 0.008) and recessive (OR = 1.39, 95%CI: 1.06–1.83, *p* = 0.018) models without adjustment. After adjustment for gender and age, the positive effect of the genotype “TT” in the two models still existed (adjusted OR = 1.61, 95%CI: 1.14–2.27, *p* = 0.007; adjusted OR = 1.41, 95%CI: 1.07–1.85, *p* = 0.015). In addition, the log-additive model showed that there was significantly increased association between rs115510139 and lung cancer risk with or without adjustment for gender and age (OR = 1.27, 95%CI: 1.07–1.50, *p* = 0.007; adjusted OR = 1.27, 95%CI: 1.07–1.51, *p* = 0.006).

**Table 2:** Significant *COL6A3* variants associated with lung cancer susceptibility

Gene	SNP	Model	Genotype	Control	Case	Unadjusted		Adjusted for Gender and Age	
						OR (95%CI)	*p*^a^-value	OR (95%CI)	*p*^b^-value
*COL6A3*	rs115510139	Codominant	A/A	131 (26.7%)	108 (21.2%)	1.00		1.00	
			A/T	232 (47.3%)	235 (46.1%)	1.23 (0.90–1.68)	0.197	1.23 (0.90–1.68)	0.196
			T/T	127 (26.0%)	167 (32.7%)	1.60 (1.13–2.25)	**0.008**	1.61 (1.14–2.27)	**0.007**
		Dominant	A/A	131 (26.7%)	108 (21.2%)	1.00	**0.040**	1.00	**0.037**
			T/A-T/T	359 (73.3%)	402 (78.8%)	1.36 (1.01–1.82)		1.36 (1.02–1.83)	
		Recessive	A/A-A/T	363 (74.0%)	343 (67.3%)	1.00	**0.018**	1.00	**0.015**
			T/T	127 (26.0%)	167 (32.7%)	1.39 (1.06–1.83)		1.41 (1.07–1.85)	
		Log-additive	–	–	–	1.27 (1.07–1.50)	**0.007**	1.27 (1.07–1.51)	**0.006**

Bold type indicates statistical significance (*p* < 0.05)

*CI* Confidence interval, *OR* Odds ratio, *SNP* Single-nucleotide polymorphism

*p*^a^: Calculated by logistic regression analysis

*p*^b^: Calculated by logistic regression analysis adjusted for gender and age

### Stratification analysis by gender

Furthermore, after a stratified analysis by gender, we found that the two variants (rs115510139 and rs3736341) were significant in males but were non-significant in females based on the allele model (Table 3). Chi-square test showed that the frequencies distribution of minor allele of the two variants were significantly different between the controls and the male patients (*p* = 0.003 and 0.038), respectively. Rs115510139-T conferred a significantly higher likelihood of lung cancer risk than the C allele. In the codominant model, the homozygous genotype “TT” of rs115510139 (adjusted OR = 1.86; 95%CI, 1.23–2.83; *p* = 0.003) increased the risk of lung cancer by 1.86-fold. There were significantly increased association between rs115510139 and lung cancer susceptibility in the dominant (adjusted OR = 1.53, 95%CI: 1.07–2.18, *p* = 0.018), recessive (adjusted OR = 1.53, 95%CI: 1.10–2.12, *p* = 0.012), and log-additive (adjusted OR = 1.37, 95%CI: 1.11–1.68, *p* = 0.003) models, respectively.

**Table 3:** Significant variants in *COL6A3* associated with lung risk in males and females

	SNP	Model	Genotype	Control (%)	Case (%)	Adjustment With Age in males		Adjustment With Age in females	
						OR (95%CI)	*p*^#^-value	OR (95%CI)	*p*^#^-value
*COL6A3*	rs115510139	Allele	A/T	–	–	**1.38 (1.11–1.70)**	**0.003** ^*^	0.92 (0.66–1.26)	0.592
		Codominant	A/A	95 (27.5)	71 (20.0)	1.00		1.00	
			A/T	163 (47.3)	165 (46.5)	1.35 (0.93–1.97)	0.118	0.83 (0.49–1.43)	0.506
			T/T	87 (25.2)	119 (33.5)	**1.86 (1.23–2.83)**	**0.003**	0.86 (0.46–1.59)	0.624
		Dominant	A/A	95 (27.5)	71 (20.0)	1.00	**0.018**	1.00	0.497
			A/T-T/T	250 (35.1)	284 (80.0)	**1.53 (1.07–2.18)**		0.84 (0.51–1.39)	
		Recessive	A/A-A/T	258 (95.3)	236 (66.5)	1.00	**0.012**	1.00	0.870
			T/T	87 (25.2)	119 (33.5)	**1.53 (1.10–2.12)**		0.96 (0.56–1.62)	
		Log-additive	–	–	–	**1.37 (1.11–1.68)**	**0.003**	0.92 (0.68–1.26)	0.604
	rs3736341	Allele	T/C	–	–	**0.77 (0.60–0.99)**	**0.038** ^*^	1.15 (0.80–1.66)	0.437
		Codominant	T/T	177 (53.5)	214 (61.7)	1.00		1.00	
			T/C	126 (38.1)	109 (31.4)	**0.69 (0.49–0.95)**	**0.025**	1.30 (0.79–2.13)	0.306
			C/C	28 (8.4)	24 (6.9)	0.71 (0.40–1.27)	0.250	1.13 (0.51–2.50)	0.766
		Dominant	T/T	177 (53.5)	214 (61.7)	1.00	**0.019**	1.00	0.331
			T/C-C/C	154 (46.5)	133 (38.3)	**0.69 (0.51–0.94)**		1.26 (0.79–2.00)	
		Recessive	T/T-T/C	303 (91.6)	323 (93.1)	1.00	0.486	1.00	0.970
			C/C	28 (8.4)	24 (6.9)	0.82 (0.46–1.44)		1.02 (0.47–2.19)	
		Log-additive	–	–	–	**0.77 (0.61–0.98)**	**0.034**	1.14 (0.81–1.61)	0.457
	rs12052971	Allele	G/A	–	–	0.93 (0.73–1.18)	0.555^*^	1.24 (0.83–1.86)	0.293
		Codominant	G/G	193 (56.6)	218 (61.6)	1.00		1.00	
			G/A	119 (34.9)	98 (27.7)	**0.72 (0.51–0.99)**	**0.048**	1.13 (0.67–1.89)	0.657
			A/A	29 (8.5)	38 (10.7)	1.19 (0.71–2.01)	0.513	1.74 (0.63–4.82)	0.284
		Dominant	G/G	193 (56.6)	218 (61.6)	1.00	0.168	1.00	0.450
			G/A-A/A	148 (43.4)	136 (38.4)	0.81 (0.60–1.09)		1.21 (0.74–1.98)	
		Recessive	G/G-G/A	312 (91.5)	316 (89.3)	1.00	0.267	1.00	0.314
			A/A	29 (8.5)	38 (10.7)	1.34 (0.80–2.23)		1.67 (0.61–4.54)	
		Log-additive	–	–	–	0.94 (0.75–1.18)	0.598	1.22 (0.83–1.81)	0.313

Bold type indicates statistical significance (*p* < 0.05)

*SNP* Single nucleotide polymorphism, *OR* Odds ratio, *95%CI* 95% confidence interval

p^*^: Calculated by Pearson χ^2^ test

*p*^#^: *p*-values were calculated by logistic regression analysis with adjustment for age

In addition, heterozygous genotype “T/C” of rs3736341 (adjusted OR = 0.69, 95%CI: 0.49–0.95, *p* = 0.025) showed a protective effect on the risk of lung cancer in the codominant model in males. And there was significant association between rs3736341 and lung cancer risk in the dominant (adjusted OR = 0.69, 95%CI: 0.51–0.94, *p* = 0.019) and log-additive (adjusted OR = 0.69, 95%CI: 0.49–0.95, *p* = 0.025) models. Genotype “G/A” of rs12052971 (adjusted OR = 0.72, 95%CI: 0.51–0.99, *p* = 0.048) showed that a significantly decreased association with genetic predisposition of lung cancer in the codominant model. But, non-significant associations were found between rs12052971 and lung cancer risk in any other models.

### Stratification analysis by the age of 61 years

We also observed the correlation between lung cancer susceptibility and *COL6A3* variants with stratification analysis by the age of 61 years (Table 4). There was not significantly decreased association between rs115510139 and lung cancer risk in the populations of more than 61 years, but it was statistically significant in less than 61-year-old populations. The variant rs115510139 was observed to increase lung cancer susceptibility in the codominant (OR = 1.84, 95%CI: 1.12–3.03, *p* = 0.016), dominant (OR = 1.56, 95%CI: 1.03–2.38, *p* = 0.037), and log-additive (OR = 1.36, 95%CI; 1.06–1.74, *p* = 0.016) models.

**Table 4:** Significant variants in *COL6A3* associated with lung susceptibility after stratified by age of 61 years

Gene	SNP	Model	Genotype	Control	Case	Age>61 years		Age ≤ 61 years	
						OR (95%CI)	*p*-value	OR (95%CI)	*p*-value
*COL6A3*	rs115510139	Codominant	A/A	131 (26.7%)	108 (21.2%)	1.00		1.00	
			A/T	232 (47.3%)	235 (46.1%)	0.77 (0.51–1.16)	0.212	1.40 (0.89–2.21)	0.143
			T/T	127 (26.0%)	167 (32.7%)	0.71 (0.43–1.16)	0.167	1.84 (1.12–3.03)	**0.016**
		Dominant	A/A	131 (26.7%)	108 (21.2%)	1.00	0.142	1.00	**0.037**
			T/A-T/T	359 (73.3%)	402 (78.8%)	0.75 (0.51–1.10)		1.56 (1.03–2.38)	
		Recessive	A/A-A/T	363 (74.0%)	343 (67.3%)	1.00	0.393	1.00	0.055
			T/T	127 (26.0%)	167 (32.7%)	0.83 (0.55–1.27)		1.48 (0.99–2.22)	
		Log-additive	–	–	–	0.84 (0.66–1.07)	0.154	1.36 (1.06–1.74)	**0.016**

Bold type indicates statistical significance (*p* < 0.05)

*SNP* Single nucleotide polymorphism, *OR* Odds ratio, *95%CI* 95% confidence interval

*p*: *p*-values were calculated by logistic regression analysis with adjustment for gender

### Pathological information analysis with lung adenocarcinoma and lung squamous carcinoma

After analysis for association with lung adenocarcinoma without adjustment (Table 5), we discovered that rs13032404 was significant in the recessive model (*p* = 0.038). In the other models, the genotype of rs13032404 had no significant relationship with lung cancer risk. When analyzed by association with lung squamous carcinoma, the increase risk of rs13032404 was apparent in the dominant model (adjusted OR = 1.58, 95%CI: 1.02–2.47, *p* = 0.042) after adjustment for gender and age. Besides, rs115510139 and rs3736341 were related to the risk of lung cancer in the four models. As for rs115510139, the significantly increased risk was observed in the codominant (adjusted OR = 1.58, 95%CI: 1.02–2.47, *p* = 0.042), dominant (adjusted OR = 1.89, 95%CI: 1.11–3.20, *p* = 0.019), recessive (adjusted OR = 1.59, 95%CI: 1.02–2.46, *p* = 0.040), and log-additive (adjusted OR = 1.48, 95%CI: 1.11–1.98, *p* = 0.007) models. Inversely, rs3736341 showed the protective effect on the lung cancer susceptibility in the codominant (adjusted OR = 0.33, 95%CI: 0.11–0.95, *p* = 0.040), dominant (adjusted OR = 0.61, 95%CI: 0.40–0.95, *p* = 0.027), and log-additive (adjusted OR = 0.63, 95%CI: 0.44–0.91, *p* = 0.012) models. In addition, we also evaluated the association between *COL6A3* SNPs and lung cancer patients with or without lymph node metastasis (Additional file 5: Table S2) as well as the clinical staging of lung cancer patients (Additional file 6: Table S3). But, the three variants did not show a significant correlation with the susceptibility of lung cancer.

**Table 5:** Significant variants in *COL6A3* associated with lung risk in patients with lung adenocarcinoma and lung squamous carcinoma

	SNP	Model	Genotype	Control (%)	Case (%)	Without Adjustment		Adjustment by gender and age	
						OR (95%CI)	*p*-value	OR (95%CI)	*p*-value
Lung adenocarcinoma									
*COL6A3*	rs13032404	Codominant	A/A	95 (27.5)	71 (20.0)	1.00		1.00	
			A/T	163 (47.3)	165 (46.5)	0.93 (0.64–1.35)	0.703	0.92 (0.63–1.34)	0.672
			T/T	87 (25.2)	119 (33.5)	1.56 (0.95–2.59)	0.768	1.46 (0.88–2.44)	0.146
		Dominant	A/A	95 (27.5)	71 (20.0)	1.00	0.018	1.00	0.869
			A/T-T/T	250 (35.1)	284 (80.0)	1.05 (0.74–1.49)		1.03 (0.72–1.46)	
		Recessive	A/A-A/T	258 (95.3)	236 (66.5)	1.00	0.038	1.00	0.076
			T/T	87 (25.2)	119 (33.5)	1.63 (0.92–2.58)		1.53 (0.96–2.44)	
		Log-additive	–	–	–	1.17 (0.92–1.50)	0.204	1.14 (0.89–1.46)	0.305
Lung Squamous Carcinoma									
*COL6A3*	rs13032404	Codominant	A/A	95 (27.5)	71 (20.0)	1.00		1.00	
			A/T	163 (47.3)	165 (46.5)	1.51 (0.96–2.36)	0.074	1.57 (0.99–2.49)	0.054
			T/T	87 (25.2)	119 (33.5)	1.56 (0.82–2.99)	0.177	1.64 (0.84–3.18)	0.147
		Dominant	A/A	95 (27.5)	71 (20.0)	1.00	0.059	1.00	**0.042**
			A/T-T/T	250 (35.1)	284 (80.0)	1.52 (0.98–2.34)		**1.58 (1.02–2.47)**	
		Recessive	A/A-A/T	258 (95.3)	236 (66.5)	1.00	0.508	1.00	0.475
			T/T	87 (25.2)	119 (33.5)	1.22 (0.68–2.18)		1.24 (0.68–2.26)	
		Log-additive	–	–	–	1.30 (0.96–1.76)	0.086	1.34 (0.98–1.82)	0.064
	rs115510139	Codominant	A/A	131 (26.7%)	108 (21.2%)	1.00		1.00	
			A/T	232 (47.3%)	235 (46.1%)	1.64 (0.94–2.84)	0.080	1.68 (0.96–2.95)	0.007
			T/T	127 (26.0%)	167 (32.7%)	**2.17 (1.21–3.89)**	**0.010**	**2.27 (1.25–4.13)**	**0.007**
		Dominant	A/A	131 (26.7%)	108 (21.2%)	1.00		1.00	
			T/A-T/T	359 (73.3%)	402 (78.8%)	**1.83 (1.09–3.07)**	**0.024**	**1.89 (1.11–3.20)**	**0.019**
		Recessive	A/A-A/T	363 (74.0%)	343 (67.3%)	1.00	**0.047**	1.00	**0.040**
			T/T	127 (26.0%)	167 (32.7%)	**1.54 (1.01–2.36)**		**1.59 (1.02–2.46)**	
		Log-additive	–	–	–	**1.45 (1.10–1.92)**	**0.010**	**1.48 (1.11–1.98)**	**0.007**
	rs3736341	Codominant	T/T	177 (53.5)	214 (61.7)	1.00		1.00	
			T/C	126 (38.1)	109 (31.4)	0.69 (0.44–1.07)	0.096	0.68 (0.43–1.07)	0.098
			C/C	28 (8.4)	24 (6.9)	**0.32 (0.11–0.91)**	**0.033**	**0.33 (0.11–0.95)**	**0.040**
		Dominant	T/T	177 (53.5)	214 (61.7)	1.00	**0.025**	1.00	**0.027**
			T/C-C/C	154 (46.5)	133 (38.3)	**0.62 (0.40–0.94)**		**0.61 (0.40–0.95)**	
		Recessive	T/T-T/C	303 (91.6)	323 (93.1)	1.00	0.057	1.00	0.068
			C/C	28 (8.4)	24 (6.9)	0.36 (0.13–1.03)		0.37 (0.13–1.07)	
		Log-additive	–	–	–	**0.63 (0.44–0.90)**	**0.010**	**0.63 (0.44–0.91)**	**0.012**

*p*-values were calculated by logistic regression analysis with adjustment for gender and age

Bold type indicates statistical significance (*p* < 0.05)

*SNP* Single nucleotide polymorphism, *OR* Odds ratio, *95%CI* 95% confidence interval

### LD and haplotype analysis

**Fig. 1:**
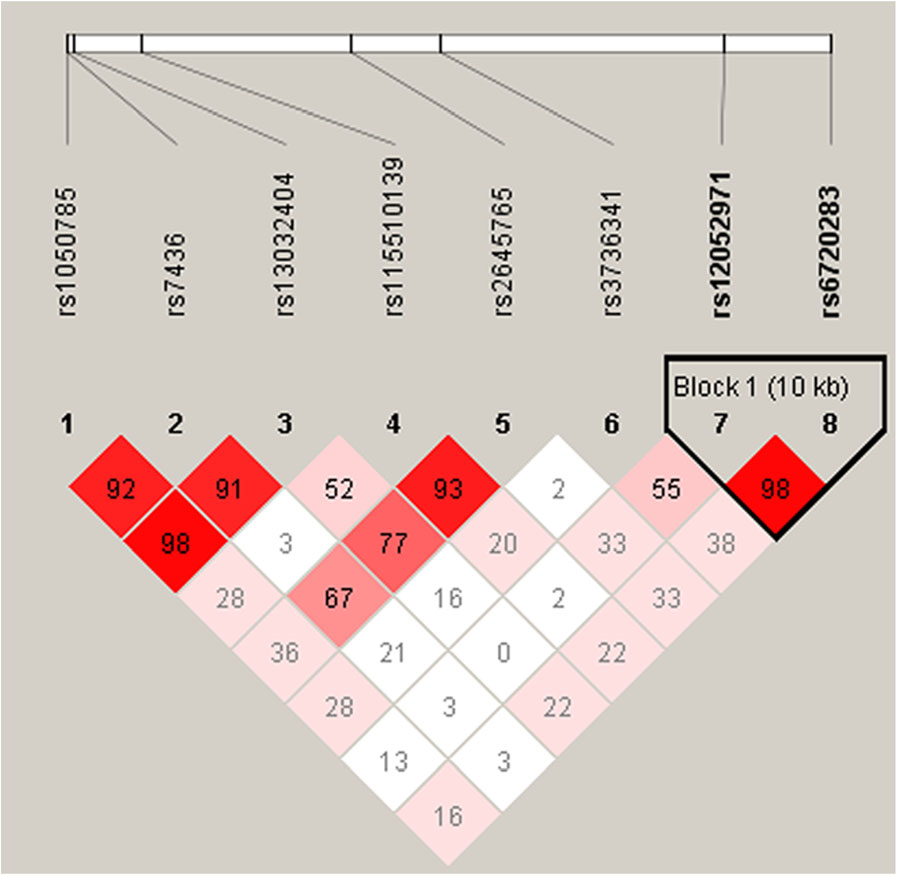
Linkage disequilibrium (LD) analysis of eight SNPs in *COL6A3.* The LD value is determined by *r*^2^ > 0.8 analyzed by Haploview software 4.2. The number in the diamonds is the LOD score of r^2^. Standard color schemes indicates different levels of LD. Bright red: LOD > 2, D’ = 1; Pink red: LOD < 2, D’ < 1; White: LOD < 2, D’ < 1

We further performed LD analysis among the eight SNPs (rs1050785, rs7436, rs13032404, rs115510139, rs2645765, rs3736341, rs12052971, rs6720283) in *COL6A3*. A strong linkage mapped to a 10-kb LD block between rs12052971 and rs6720283 was found (Fig. 1). Unfortunately, there were no statistically significant differences between patients and controls among the *COL6A3* haplotypes (Additional file 7: Table S4).

## Discussion

In the present case-control study, we investigated the connections of eight variants of *COL6A3* and lung cancer risk in a Han population of China. Our results firstly revealed that rs115510139 was significantly associated with lung cancer risk. And when stratified analysis by clinical information, rs13032404, rs115510139, rs3736341, rs12052971 were found to be related with lung cancer.

The *COL6A3* gene encodes the alpha-3 chain of type VI collagen, one of the three alpha chains of type VI collagen. Type VI collagen is structured as a trimer composed of three different alpha chains: alpha-1(VI), alpha-2(VI), and alpha-3(VI). The protein is a ubiquitous extracellular matrix protein and usually found in most connective tissues, including muscle, skin, tendon, and vessels. It is now clear that type VI collagen has vital role and can suppress apoptosis and oxidative damage, affect metabolic level, and enhance cell growth. Recent researches focused on genetic studies of lung cancer and revealed some achievements [11, 12]. Because gene mutations in *COL6A1*, *COL6A2* and *COL6A3* have been shown to result in muscular dystrophy, which indicated that collagen VI is particularly necessary for the vitality of skeletal muscle. Further, the article by Lampe AK et al. [8] about collagen VI related muscle disorders describes that mutations in the collagen genes (*COL6A1*, *COL6A2*, and *COL6A3*) can give rise to Bethlem myopathy (BM) and Ullrich congenital muscular dystrophy (UCMD). The recessive *COL6A3* mutations (p.R3043H and p.P3082R) can cause neurological disorder early-onset isolated dystonia [11, 12]. *COL6A3* SNPs were also found to confer susceptibility to early-onset dyslipidemia [13].

Not only that, inhibiting COL6A3 expression was related to insulin resistance and adipose tissue inflammation via suppression of the induction of monocyte chemoattractant protein (MCP1) [14]. And COL6A3 was highly expressed in pancreatic ductal adenocarcinoma (PDA) tissue. In serum levels, its expression was higher associated with perineural invasion and cigarette smoking. And significantly upregulated expression of multiple genes (including COL6A2, and COL6A3) were found in adamantinomatous craniopharyngioma tumor samples [15]. Ran Ao et al. reported that silencing of COL6A3 can inhibit gastric cancer cell proliferation, migration, invasion, and apoptosis by the PI3k-Akt signaling pathway [16]. In addition, COL6A3 played the clinical relevance in the development of colorectal cancer validated by silico analysis of cell type-specific gene expression and COL6A3 knockout experiments [17]. In the database of UALCAN (http://ualcan.path.uab.edu/index.html) (Additional file 1: Figure S1 and Additional file 2: Figure S2), COL6A3 expression indicated a significant difference between normal lung tissues and lung adenocarcinoma- or lung squamous cell carcinoma tumor-samples. Further, the correlations of survival rates with the expression of COL6A3 in lung cancer illustrated that lung cancer patients with higher COL6A3 expression had a lower survival rates (HR =1.32, 95%CI: 1.11–1.58, log-rank *p* = 0.0018, Additional file 3: Figure S3) shown in the database of Kaplan-Meier Plotter (http://kmplot.com/analysis/index.php?p=service&cancer=gastric). Hence, COL6A3 was indeed involved in the process of many cancers.

In the article, our results demonstrated that *COL6A3* gene was involved in the progress of lung cancer. However, the overall information about the association between *COL6A3* polymorphisms and lung cancer risk was few. Thus, a larger sample size and more in-depth analyses will be needed to verify the above results.

## Conclusion

In our results, we observed that rs115510139 was linked to an increased risk of lung cancer in the models analysis with or without adjustment for gender and age. After stratification analysis by gender and age of 61 years, rs115510139 was still associated with lung cancer risk. When analyzed for the association with lung squamous carcinoma, rs13032404, rs115510139 and rs3736341 were related to the risk of lung cancer. Our findings showed potential associations between *COL6A3* polymorphisms and lung cancer risk, which may contribute to the identification of lung cancer patients in a Chinese populations.

## Additional files

Additional file 1: **Figure S1.** Expression of *COL6A3* in normal lung tissues and lung adenocarcinoma (LUAD) tissues. There was significant difference between normal lung tissues (*n* = 59) and LUAD tissues (*n* = 515) (*p* < 0.05). Lung adenocarcinoma, LUAD. (TIF 1549 kb)

Additional file 2: **Figure S2.** Expression of *COL6A3* in normal lung tissues and lung squamous cell carcinoma (LUSC) tissues. There was significant difference between normal lung tissues (*n* = 52) and LUSC tissues (*n* = 503) (*p* < 0.05). Lung squamous cell carcinoma, LUSC. (TIF 1551 kb)

Additional file 3: **Figure S3.** The association between COL6A3 expression and survival rate in lung cancer patients. Lung cancer patients with higher COL6A3 expression had a lower survival rates shown in the database of Kaplan-Meier Plotter. (TIF 910 kb)

Additional file 4: **Table S1.** PCR primers for amplification and extension of loci used in this study. (DOCX 17 kb)

Additional file 5: **Table S2.** Significant variants in *COL6A3* associated with lung cancer risk in patients with lymph node metastasis. (DOCX 26 kb)

Additional file 6: **Table S3.** Significant variants in *COL6A3* associated with the stages of lung cancer patients. (DOCX 25 kb)

Additional file 7: **Table S4.** *COL6A3* haplotypes frequencies associated with lung cancer risk. (DOCX 18 kb)

Additional file 8: **Table S5.** In silico analysis for SNP function annotation. (DOCX 20 kb)

## Abbreviations

95%CI: 95% confidence interval
CMDs: Congenital muscular dystrophies
*COL6A3*: Collagen Type VI Alpha 3 Chain
HWE: Hardy-Weinberg Equilibrium
LD: Linkage disequilibrium
NSCLC: Non-small cell lung cancer
OR: Odds ratio
SCLC: Small cell lung cancer
SNPs: Single nucleotide polymorphisms
