# Supplementary figures and images for: COL6A3 polymorphisms were associated with lung cancer risk in a Chinese population
Source: Respir Res. 2019 Jul 8;20:143. doi: 10.1186/s12931-019-1114-y (PMC6615180; doi:10.1186/s12931-019-1114-y)

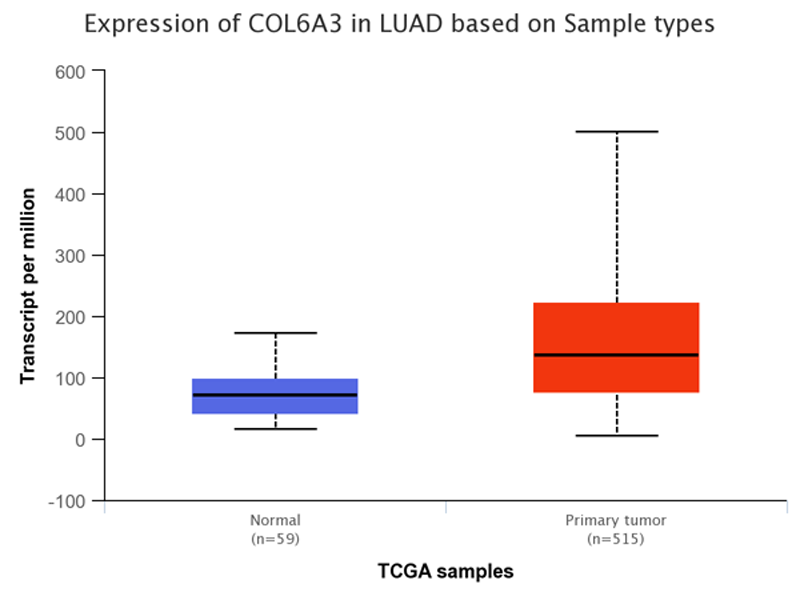

Supplement: Supplementary file 1 — Figure S1. Expression of COL6A3 in normal lung tissues and lung adenocarcinoma (LUAD) tissues. There was significant difference between normal lung tissues (n = 59) and LUAD tissues (n = 515) (p < 0.05). Lung adenocarcinoma, LUAD. (TIF 1549 kb) [file 12931_2019_1114_MOESM1_ESM.tif]

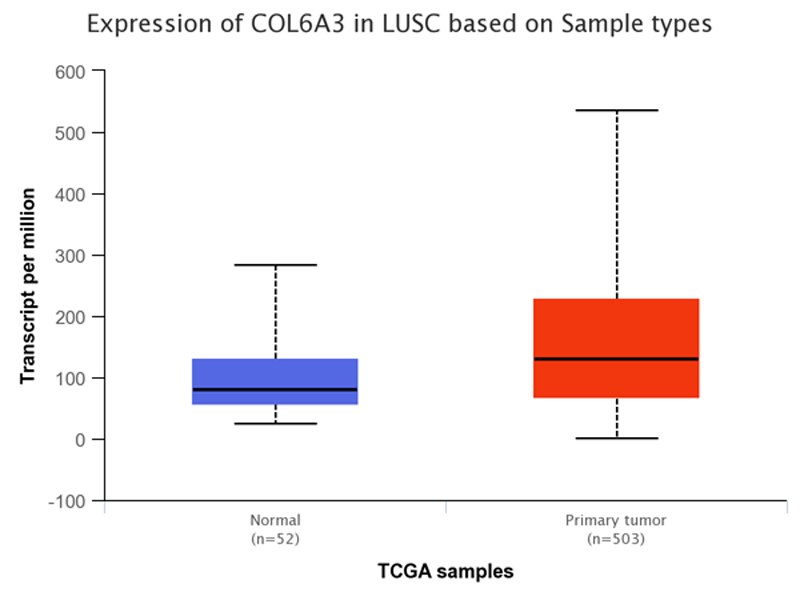

Supplement: Supplementary file 2 — Figure S2. Expression of COL6A3 in normal lung tissues and lung squamous cell carcinoma (LUSC) tissues. There was significant difference between normal lung tissues (n = 52) and LUSC tissues (n = 503) (p < 0.05). Lung squamous cell carcinoma, LUSC. (TIF 1551 kb) [file 12931_2019_1114_MOESM2_ESM.tif]

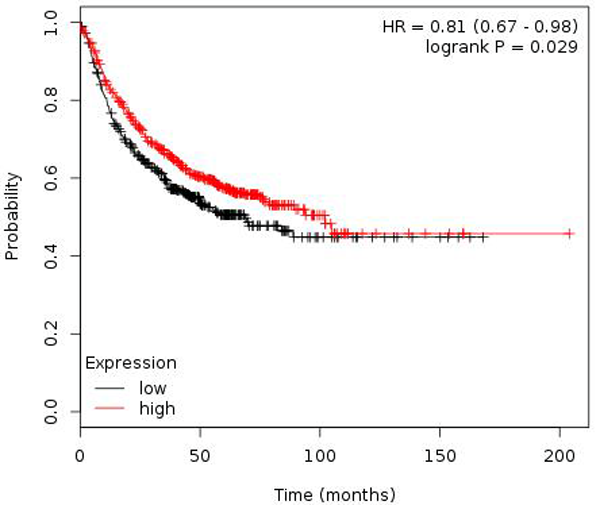

Supplement: Supplementary file 3 — Figure S3. The association between COL6A3 expression and survival rate in lung cancer patients. Lung cancer patients with higher COL6A3 expression had a lower survival rates shown in the database of Kaplan-Meier Plotter. (TIF 910 kb) [file 12931_2019_1114_MOESM3_ESM.tif]
