# Supplementary material for: COL6A3 polymorphisms were associated with lung cancer risk in a Chinese population
Source: Respir Res. 2019 Jul 8;20:143. doi: 10.1186/s12931-019-1114-y (PMC6615180; doi:10.1186/s12931-019-1114-y)
Supplement: Supplementary file 4 — Table S1. PCR primers for amplification and extension of loci used in this study. (DOCX 17 kb) [file 12931_2019_1114_MOESM4_ESM.docx]

| SNP_ID | Allele | 1st-PCR primer sequence | 2nd-PCR primer sequence | UEP sequence |
| --- | --- | --- | --- | --- |
| rs1050785 | G/T | ACGTTGGATGGGCATTCCTCTAATGTTGTG | ACGTTGGATGAATTAAATCCCCTCCCTCC | CCTCCCTCCAGCACACA |
| rs7436 | A/T | ACGTTGGATGCCCTTAATCTATGTGCACCG | ACGTTGGATGCACAACATTAGAGGAATGC | CAAAAATATTCTCTATTACAACTTTTT |
| rs13032404 | A/G | ACGTTGGATGCCATCACACTGATGACTCCG | ACGTTGGATGCTTTTCCTCTCTTCTCTCGG | aacatGGCTCCTCACCTCACCC |
| rs115510139 | A/T | ACGTTGGATGCAGAGATCAATTATGCAGGG | ACGTTGGATGTTCCTTCCCAGGGTGAAGAC | AGACATAAATTACCTTTTTTTTTTT |
| rs2645765 | A/G | ACGTTGGATGCTGTAGCCACACAACAGAGT | ACGTTGGATGGGAGCATCATGTTCTTGAGC | AGCACTTCATTCCTTTACATG |
| rs3736341 | C/T | ACGTTGGATGGATCTCAAGAAATGCCCAGC | ACGTTGGATGAGGGACTTCATCTCACACCG | CATCCGATTTTGACCCTT |
| rs12052971 | A/G | ACGTTGGATGCCAGGGATGTCAAAGGAAAC | ACGTTGGATGCTGGTTTTCTGCTTCTATGC | aTATGCTTTATGTATCACATCCTCT |
| rs6720283 | A/G | ACGTTGGATGCTTTGAGATCCTGTTTTCAC | ACGTTGGATGCTCCATCGTATGAATGTTCC | tcagATAATTCATCTATCCCACTTTTG |

Additional file 4: Table S1. PCR primers for amplification and extension of loci used in this study

PCR: polymerase chain reaction; SNP: single-nucleotide polymorphism; UEP: unextended mini-sequencing primer.
