# Supplementary material for: COL6A3 polymorphisms were associated with lung cancer risk in a Chinese population
Source: Respir Res. 2019 Jul 8;20:143. doi: 10.1186/s12931-019-1114-y (PMC6615180; doi:10.1186/s12931-019-1114-y)
Supplement: Supplementary file 5 — Table S2. Significant variants in COL6A3 associated with lung cancer risk in patients with lymph node metastasis. (DOCX 26 kb) [file 12931_2019_1114_MOESM5_ESM.docx]

Additional file 5: Table S2. Significant variants in *COL6A3* associated with lung cancer risk in patients with lymph node metastasis

|  | SNP | Model | Genotype | Non-metastasis(%) | Metastasis (%) | Without Adjustment | | Adjustment by gender and age | |
| --- | --- | --- | --- | --- | --- | --- | --- | --- | --- |
|  |  |  |  |  |  | OR (95%CI) | *p*-value | OR (95%CI) | *p*-value |
|  | rs13032404 | Codominant | A/A | 15 (17.9) | 26 (12.1) | 1.00 |  | 1.00 |  |
|  |  |  | A/G | 37 (44.0) | 107 (49.8) | 1.13 (0.65-1.96) | 0.669 | 1.16 (0.66-2.02) | 0.610 |
|  |  |  | G/G | 32 (38.1) | 82 (38.1) | 0.68 (0.32-1.44) | 0.311 | 0.68 (0.32-1.47) | 0.330 |
|  |  | Dominant | A/A | 15 (17.9) | 26 (12.1) | 1.00 | 0.994 | 1.00 | 0.939 |
|  |  |  | A/G-G/G | 69 (82.1) | 189 (87.9) | 1.00 (0.59-1.68) |  | 1.02 (0.61-1.72) |  |
|  |  | Recessive | A/A-A/G | 52 (61.9) | 133 (61.9) | 1.00 | 0.195 | 1.00 | 0.199 |
|  |  |  | G/G | 32 (38.1) | 82 (38.1) | 0.63 (0.32-1.27) |  | 0.63 (0.31-1.27) |  |
|  |  | Log-additive | --- | --- | --- | 0.88 (0.61-1.28) | 0.505 | 0.89 (0.61-1.30) | 0.548 |
|  | rs115510139 | Codominant | A/A | 16 (19.0%) | 50 (21.2%) | 1.00 |  | 1.00 |  |
|  |  |  | A/T | 43 (51.2%) | 98 (46.1%) | 0.85 (0.47-1.52) | 0.586 | 0.86 (0.48-1.54) | 0.609 |
|  |  |  | T/T | 25 (29.8%) | 67 (32.7%) | 1.17 (0.56-2.41) | 0.679 | 1.16 (0.56-2.40) | 0.696 |
|  |  | Dominant | A/A | 16 (19.0%) | 50 (21.2%) | 1.00 | 0.814 | 1.00 | 0.824 |
| *COL6A3* |  |  | T/A-T/T | 68 (81.0%) | 165 (78.8%) | 0.94 (0.54-1.62) |  | 0.94 (0.54-1.63) |  |
|  |  | Recessive | A/A-A/T | 59 (70.2%) | 148 (67.3%) | 1.00 | 0.431 | 1.00 | 0.460 |
|  |  |  | T/T | 25 (29.8%) | 67 (32.7%) | 1.29 (0.69-2.42) |  | 1.27 (0.67-2.40) |  |
|  |  | Log-additive | --- | --- | --- | 1.06 (0.74-1.50) | 0.762 | 1.05 (0.74-1.50) | 0.778 |
|  | rs3736341 | Codominant | C/C | 4 (4.9) | 17 (8.4) | 1.00 |  | 1.00 |  |
|  |  |  | C/T | 28 (34.1) | 79 (38.9) | 1.25 (0.72-2.15) | 0.425 | 1.24 (0.72-2.15) | 0.436 |
|  |  |  | T/T | 50 (61.0) | 113 (55.7) | 1.88 (0.60-5.87) | 0.277 | 1.80 (0.57-5.64) | 0.315 |
|  |  | Dominant | C/C | 4 (4.9) | 17 (8.4) | 1.00 | 0.286 | 1.00 | 0.307 |
|  |  |  | C/T-T/T | 78 (95.1) | 192 (94.6) | 1.33 (0.79-2.23) |  | 1.31 (0.78-2.22) |  |
|  |  | Recessive | C/C-C/T | 32 (39.0) | 96 (47.3) | 1.00 |  | 1.00 |  |
|  |  |  | T/T | 50 (61.0) | 113 (55.7) | 1.73 (0.56-5.29) | 0.339 | 1.65 (0.54-5.10) | 0.381 |
|  |  | Log-additive | --- | --- | --- | 1.31 (0.86-1.99) | 0.215 | 1.29 (0.84-1.97) | 0.242 |

SNP: Single nucleotide polymorphism; OR: odds ratio; 95%CI: 95% confidence interval.

*p*-values were calculated by logistic regression analysis with adjustment for gender and age.
