# Supplementary material for: COL6A3 polymorphisms were associated with lung cancer risk in a Chinese population
Source: Respir Res. 2019 Jul 8;20:143. doi: 10.1186/s12931-019-1114-y (PMC6615180; doi:10.1186/s12931-019-1114-y)
Supplement: Supplementary file 6 — Table S3. Significant variants in COL6A3 associated with the stages of lung cancer patients. (DOCX 25 kb) [file 12931_2019_1114_MOESM6_ESM.docx]

Additional file 6: Table S3. Significant variants in *COL6A3* associated with the stages of lung cancer patients

|  | SNP | Model | Genotype | T1-T2 (%) | T3-T4 (%) | Without Adjustment | | Adjustment by gender and age | |
| --- | --- | --- | --- | --- | --- | --- | --- | --- | --- |
|  |  |  |  |  |  | OR (95%CI) | *p*-value | OR (95%CI) | *p*-value |
|  | rs13032404 | Codominant | A/A | 95 (27.5) | 71 (20.0) | 1.00 |  | 1.00 |  |
|  |  |  | A/T | 163 (47.3) | 165 (46.5) | 1.17 (0.69-2.00) | 0.555 | 1.19 (0.70-2.03) | 0.521 |
|  |  |  | T/T | 87 (25.2) | 119 (33.5) | 1.15 (0.54-2.45) | 0.715 | 1.12 (0.53-2.40) | 0.764 |
|  |  | Dominant | A/A | 95 (27.5) | 71 (20.0) | 1.00 | 0.544 | 1.00 | 0.531 |
|  |  |  | A/T-T/T | 250 (35.1) | 284 (80.0) | 1.17 (0.71-1.93) |  | 1.18 (0.71-1.95) |  |
|  |  | Recessive | A/A-A/T | 258 (95.3) | 236 (66.5) | 1.00 | 0.883 | 1.00 | 0.952 |
|  |  |  | T/T | 87 (25.2) | 119 (33.5) | 1.05 (0.52-2.12) |  | 1.02 (0.51-2.07) |  |
|  |  | Log-additive | --- | --- | --- | 1.10 (0.77-1.57) | 0.614 | 1.09 (0.76-1.57) | 0.635 |
|  | rs115510139 | Codominant | A/A | 131 (26.7%) | 108 (21.2%) | 1.00 |  | 1.00 |  |
|  |  |  | A/T | 232 (47.3%) | 235 (46.1%) | 1.52 (0.83-2.79) | 0.174 | 1.52 (0.82-2.79) | 0.180 |
|  |  |  | T/T | 127 (26.0%) | 167 (32.7%) | 1.86 (0.95-3.65) | 0.071 | 1.87 (0.95-3.68) | 0.069 |
|  |  | Dominant | A/A | 131 (26.7%) | 108 (21.2%) | 1.00 | 0.083 | 1.00 | 0.084 |
| *COL6A3* |  |  | T/A-T/T | 359 (73.3%) | 402 (78.8%) | 1.65 (0.94-2.89) |  | 1.65 (0.94-2.90) |  |
|  |  | Recessive | A/A-A/T | 363 (74.0%) | 343 (67.3%) | 1.00 | 0.222 | 1.00 | 0.210 |
|  |  |  | T/T | 127 (26.0%) | 167 (32.7%) | 1.41 (0.81-2.44) |  | 1.42 (0.82-2.47) |  |
|  |  | Log-additive | --- | --- | --- | 1.36 (0.97-1.92) | 0.076 | 1.37 (0.97-1.92) | 0.073 |
|  | rs3736341 | Codominant | T/T | 177 (53.5) | 214 (61.7) | 1.00 |  | 1.00 |  |
|  |  |  | T/C | 126 (38.1) | 109 (31.4) | 0.77 (0.46-1.31) | 0.337 | 0.76 (0.45-1.29) | 0.309 |
|  |  |  | C/C | 28 (8.4) | 24 (6.9) | 1.75 (0.57-5.32) | 0.325 | 1.71 (0.56-5.22) | 0.346 |
|  |  | Dominant | T/T | 177 (53.5) | 214 (61.7) | 1.00 | 0.615 | 1.00 | 0.568 |
|  |  |  | T/C-C/C | 154 (46.5) | 133 (38.3) | 0.88 (0.53-1.45) |  | 0.86 (0.52-1.43) |  |
|  |  | Recessive | T/T-T/C | 303 (91.6) | 323 (93.1) | 1.00 |  | 1.00 |  |
|  |  |  | C/C | 28 (8.4) | 24 (6.9) | 1.93 (0.65-5.76) | 0.237 | 1.91 (0.64-5.69) | 0.248 |
|  |  | Log-additive | --- | --- | --- | 1.02 (0.69-1.51) | 0.904 | 1.01 (0.68-1.50) | 0.955 |

SNP: Single nucleotide polymorphism; OR: odds ratio; 95%CI: 95% confidence interval.

*p*-values were calculated by logistic regression analysis with adjustment for gender and age.
