# Supplementary material for: COL6A3 polymorphisms were associated with lung cancer risk in a Chinese population
Source: Respir Res. 2019 Jul 8;20:143. doi: 10.1186/s12931-019-1114-y (PMC6615180; doi:10.1186/s12931-019-1114-y)
Supplement: Supplementary file 7 — Table S4. COL6A3 haplotypes frequencies associated with lung cancer risk. (DOCX 18 kb) [file 12931_2019_1114_MOESM7_ESM.docx]

Additional file 7: Table S4. *COL6A3* haplotypes frequencies associated with lung cancer risk

| SNP_ID | Haplotype | Freq | | Unadjusted | | Adjusted with age and gender | |
| --- | --- | --- | --- | --- | --- | --- | --- |
|  |  | Case | Control | OR (95%CI) | *p*^a^ | OR (95%CI) | *p*^b^ |
| rs12052971\|rs6720283 | AG | 0.751 | 0.754 | 0.98 (0.81-1.19) | 0.853 | 0.98 (0.81-1.19) | 0.818 |
|  | GG | 0.209 | 0.189 | 1.13 (0.91-1.39) | 0.281 | 1.13 (0.91-1.39) | 0.284 |
|  | GA | 0.541 | 0.563 | 0.92 (0.77-1.10) | 0.354 | 0.92 (0.77-1.10) | 0.332 |

CI: Confidence interval; Freq: Frequency; OR: Odds ratio; SNP: Single nucleotide polymorphism.

*p*^a^ values were calculated by logistic regression analysis without adjusted.

*p*^b^ values were calculated by logistic regression analysis after adjusted for gender and age.
