# Supplementary material for: COL6A3 polymorphisms were associated with lung cancer risk in a Chinese population
Source: Respir Res. 2019 Jul 8;20:143. doi: 10.1186/s12931-019-1114-y (PMC6615180; doi:10.1186/s12931-019-1114-y)
Supplement: Supplementary file 8 — Table S5. In silico analysis for SNP function annotation. (DOCX 20 kb) [file 12931_2019_1114_MOESM8_ESM.docx]

Additional file 8: Table S5 In silico analysis for SNP function annotation

| SNP | Chromosome | Alleles  A<B | Gene | Regulome  DB Score | HaploReg |
| --- | --- | --- | --- | --- | --- |
|  |  |  |  |  |  |
| rs1050785 | Chr2 | G/T | *COL6A3* | 6 | Motifs changed |
| rs7436 | Chr2 | A/T | *COL6A3* | 6 | DNAse, Motifs changed |
| rs13032404 | Chr2 | A/G | *COL6A3* | 5 | DNAse, Motifs changed |
| rs115510139 | Chr2 | A/T | *COL6A3* | 6 | Motifs changed |
| rs2645765 | Chr2 | A/G | *COL6A3* | 5 | - |
| rs3736341 | Chr2 | C/T | *COL6A3* | 3a | Motifs changed |
| rs12052971 | Chr2 | A/G | *COL6A3* | 5 | DNAse, Motifs changed |
| rs6720283 | Chr2 | A/G | *COL6A3* | 5 | - |

eQTL: expression quantitative trait loci; SNP: single-nucleotide polymorphism.

3a indicates that the variant is less likely to affect binding.

5 indicates that the variant has minimal binding evidence.

6 indicates that the variant has minimal binding evidence.
